# The Effect of Palm Oil-Based Hybrid Oils as Green Multifunctional Oils on the Properties of Elastomer Composites

**DOI:** 10.3390/polym10091045

**Published:** 2018-09-19

**Authors:** Sung Ho Song

**Affiliations:** Division of Advanced Materials Engineering, Kongju National University, Chungnam 330-717, Korea; shsong805@gmail.com; Tel.: +82-041-521-9379

**Keywords:** styrene butadiene rubber, palm oil, hybrid oils, elastomer composites

## Abstract

Hybrid oils in an elastomer matrix provide superior physical and chemical properties over conventional elastomer composites. In this study, we investigated the possibility of utilizing palm-based hybrid oil as a processing oil, with various other added oils such as methylester, palm monoglyceride and dammar, and their effects on the curing characteristics, mechanical, abrasion resistance and heat build-up properties of elastomer composites. The elastomer composites with the hybrid oils exhibit remarkable improvements in mechanical properties such as modulus, tensile strength, elongation and toughness, which were ascribed to the enhanced dispersion of the fillers in the elastomer matrix. Also, the hybrid oils in the elastomer matrix showed outstanding heat build-up, abrasion and rebound resilience properties at low loadings. The synergistic effects in the elastomer matrix achieved by the hybridization of palm oil and other oils represent a significant contribution to advanced, stronger elastomer composites in various applications.

## 1. Introduction

Elastomers, which are polymers with viscoelastic properties, are considered to be excellent host matrixes for composite materials, and are widely used in practical industry applications [1,2,3,4,5]. The conventional elastomer compound contains an elastomer, filler, activator, sulfur, accelerator, and processing aids to enhance its properties in the tire industry. Among these, the role of the processing aid or physical plasticizer is to modify the physical properties of the elastomer composites. Types of plasticizers include mineral oils, synthetic esters and some natural products such as animal glue and wood rosin [6].

Oil products are used as plasticizers for plastics to make them flexible or plastic during processing. Generally, petroleum and vegetable oils are added to elastomer compounds as softeners to facilitate their processing for extrusion and calendaring and to enhance the addition and dispersion of the other compounding ingredients. Also, stickiness is improved by adding softeners, which is essential for many applications, such as tires, seals, belts and impact bumpers. In the tire industry in particular, petroleum oils such as aromatic, naphthenic and paraffin oils are used as process aids [7]. However, given the depletion of petroleum resources and increasing incidence of cancer it has become necessary to find alternative oils [8].

Vegetable oil esters have a higher molecular weight and free fatty acids as coactivators compared to conventional synthetic esters. Almost all vegetable oils are potential antioxidants and can protect the elastomers from oxidation. Therefore, vegetable oils can be alternative multifunctional ingredients in elastomer composites [8,9,10,11,12].

As a processing aid, rice oil has been used in styrene butadiene rubbers [9], polychloroprene (CR) [7] and acrylonitrile butadiene (NBR). Also, castor oil has been added in nitrocellulose, natural rubber (NR), and polystyrene rubber [10,11]. Rice-bran oil has been used as a processing aid in acrylonitrile-butadiene (NBR), polychloroprene (CR) [7] and styrene-butadiene (SBR) rubbers [9]. Kukreja and coworkers reported that acrylonitrile butadiene elastomer composites with dehydrated castor oil substituted dioctylphthalate showed increased plasticizing efficiency and aging resistance [12]. Also, Asharf and coworkers found that the mechanical properties of poly(methyl methacrylate) (PMMA) were improved by adding dehydrated castor oil [13]. Soy bean oil was used as a plasticizer in NR [14] and blown soya bean oil was used as plasticizer in ester gums [10].

In recent years, hybrid oils have been used as a multipurpose additive in elastomers to control its processability, cure time, and mechanical properties. Raju and coworkers reported that NBR composites with linseed oil improved their curing and mechanical properties [15]. The reinforcement of elastomers using the synergetic effect of two different oils holds great potential interest, but few studies have been devoted to similar synergetic effects in elastomers.

In this study we conducted hybridizations of palm oil and other oils such as methylester, palm monoglyceride and dammar to form hybrid oil additives which showed versatile and customized properties that were different to those of the individual oils. The palm oil-based hybrid oils appear to be promising elastomer additives because they promoted high modulus, tensile strength, elongation and toughness. Also, the improved dispersion of fillers in the elastomer matrix produced a remarkable synergetic effect which significantly enhanced abrasion resistance, heat build-up and rebound resilience properties. Based on these results, we can confirm that palm oil-based hybrid oils may be used as multipurpose additives in elastomer nanocomposites. 

## 2. Experimental Section

### 2.1. Materials 

The matrix material selected was styrene butadiene rubber (SBR 1500, Kumho petrochem Co. Ltd., Yeosu, Korea) consisting of styrene of 23% and butadiene of 77%. Carbon black (N-330, OCI Co. Ltd., Gwangyang, Korea), *N*-tert-butyl-benzothiazole sulfonamide (TBBS, Shangdong Shanxian Co. Ltd., Shangdong, China) were used and zinc oxide (ZnO), stearic acid (S/A) and sulfur were purchased from Sigma-Aldrich (St. Louis, MO, USA). Palm oil, metylester, PMG and dammer were purchased from Kumho petrochem Co. Ltd., Yeosu, Korea.

### 2.2. Preparation of Elastomer Composites

The hybrid oils/SBR nanocomposites were prepared by following standard procedures in Table 1. Firstly, the hybrid oils were mixed palm oil (OTG) and other oils (methylester (OTG/M), palm monoglyceride (OTG/P), dammar (OTG/D)) in the same weight ratio (1:1) by the stirring method at 70 °C. The SBR was mixed with 50 phr (parts per hundred rubber by weight) carbon black with 3 phr hybrid oils in a Banbury mixer at a rotor speed of 60 rpm. The additives and vulcanization agents were added at the end so that a curing process of the mixture could be started. The compounds were placed in the aluminum mold and cured at 160 °C for *T*_90_ by rheometer under pressure. The formulations of the diverse oils/SBR composites are summarized in Table 1.

### 2.3. Swelling Study and Crosslinking Density

Swelling tests were carried out by soaking a specific weight of elastomer composites in toluene at room temperature for 24 h. The equilibrium swelling in toluene (Q%) was determined according to the standard method (ASTM D471-06). The equilibrium swelling Q% could be calculated according to the following equation Equation (1):

Q = [(*W*_s_ − *W*_d_)/*W*_d_]·100% (1)

where *W*_s_: the weight of the swelled specimen, *W*_d_: the weight of the dried specimen.

The crosslinking density (V), mol/cm^3^ of SBR was determined on the basis of solvent-swelling measurements (toluene solvent for 24 h at 25 ± 1 °C) using the Flory–Rehner equation in Equations (2) and (3) [16,17]

V = 0.5/*M*c (2)

where *M*c: the molecular weight between crosslinks (g/mol).

(3) $$Mc=[\frac{-\rho Vs\left(Vr^{1/3}-\frac{2Vr}{f}\right)}{\left[\ln\left(1-Vr\right)+Vr+\chi Vr^{2}\right]}]$$

where ρ: the density of rubbers, cs: the molar volume of the solvent (toluene), f: network functionality, *χ*: the interaction parameter of elastomers, *V*_r_: the volume fraction of swollen elastomer that can be obtained from the mass and density of elastomer samples and the solvent.

### 2.4. Characterization

Scanning electron microscopy (SEM, JEOL JSM-6490LV, Tokyo, Japan) was used to observe the morphology of the fractured surface of elastomer composites. The specimens were fractured in liquid nitrogen and the cross section of samples were coated by gold using a sputtering process. Curing characteristics were measured over 30 min periods at 160 °C using a moving-die rheometer (DRM-100(LP-171)). Tensile tests were carried out in an Instron tensile machine (Instron Co., Cambridge, UK) at crosshead speed of 500 mm/min. The dumbbell shape samples were 100 mm in thickness and 5 mm in width. At least four tests were carried out for each case. Dynamic tests were conducted using a dynamic mechanical analyzer (model DMA 50N01Db; Metravib, Bruggen, German) in tensile mode. The temperature dependence (temperature sweep) of the storage modulus and the loss factor (tan δ, defined as the ratio of the loss modulus to the storage modulus) were measured from −20 to 100 °C at a heating rate of 3 °C/min and a frequency of 10 Hz. The fatigue properties of composites were characterized by using Demmattia (UESHIMA, New York, USA). 

## 3. Results and Discussion

**Figure 1:**
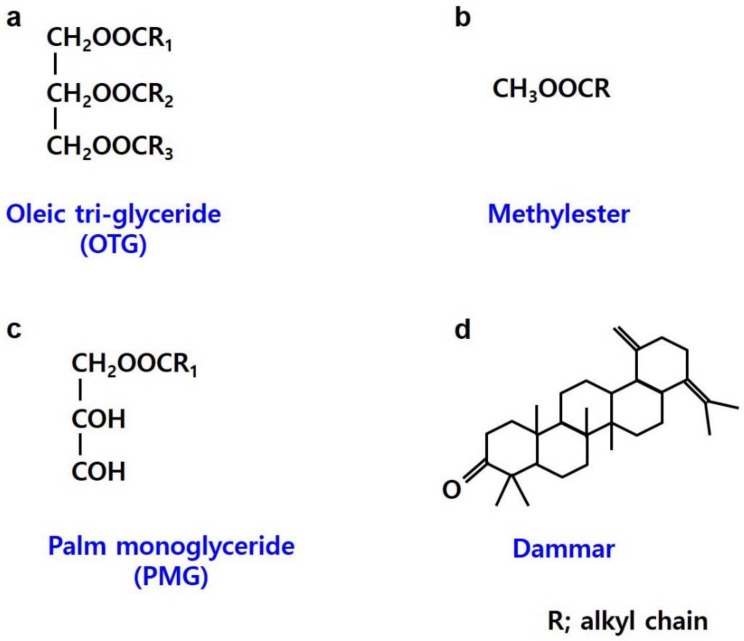
Schematic diagram of diverse oils (**a**) Schematic diagram of palm oil (oleic tri-glyceride). (**b**) Schematic diagram of methylester oil. (**c**) Schematic diagram of palm monoglyceride oil. (**d**) Schematic diagram of dammar oil.

Composites of carbon black and elastomers produced by Banbury internal mixing were prepared with palm oil-based hybrid oils for comparison with composites with unmodified palm oil (control). The overall oils used to fabricate the hybrid oils are illustrated in Figure 1. The hybrid oils of fabricated palm oil (OTG) and other oils (methylester (OTG/M), palm monoglyceride (OTG/P), dammar (OTG/D) were mixed in the same ratio (1:1) based on palm oil at 70 °C by stirring as shown in Figure 1.

**Figure 2:**
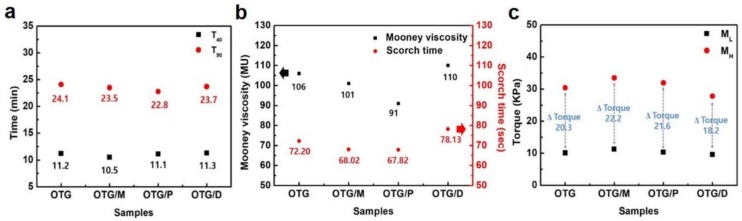
Curing properties of elastomer composites. (**a**) Curing properties of elastomer composites with hybrid oils. (**b**) Mooney viscosity of the elastomer composites with hybrid oils. (**c**) Torque of elastomer composites with hybrid oils.

The processability of composites is important and was determined by evaluating cure characteristics such as cure time, scorch time, maximum torque and minimum torque. The cure characteristics based on scorch time (t_40_) and cure time (t_90_) of different oil-filled elastomer vulcanizates are shown in Figure 2a. The values of the scorch and cure times of the elastomer composites with various oils did not exhibit any apparent difference because small quantities of oil were added without changing the thermal conductivity, as shown in Figure 2b. 

Also, Mooney viscosity is commonly used as an indicator of processability for elastomer materials. The OTG/P reduced the Mooney viscosity and apparent shear viscosity of the elastomer composites, which facilitated the manufacturing process of the elastomer composites, as indicated in Figure 2b. 

**Figure 3:**
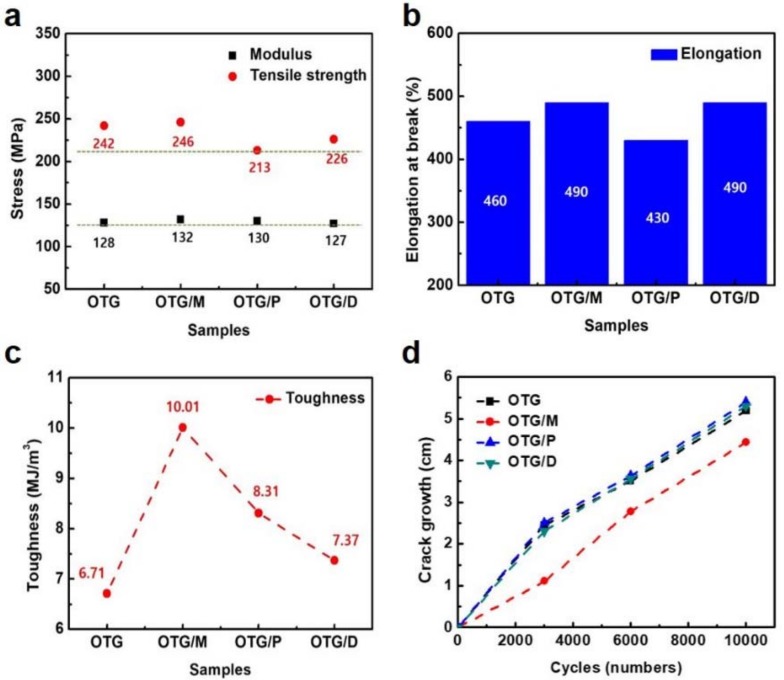
Mechanical properties of elastomer composites. (**a**) Elastic modulus and tensile strength of the elastomer composites with hybrid oils. (**b**) Elongation of the elastomer composites with hybrid oils. (**c**) Toughness of the elastomer composites with hybrid oils. (**d**) Fatigue properties (fatigue crack growth) of the elastomer composites with hybrid oils.

A comparison of torque in the elastomer composites with different embedded oils is presented in Figure 2c. The *M*_H_ − *M*_L_ (∆ Torque) value (22.2 MPa) of the SBR composites with OTG/M, the crosslink density of vulcanization, was meaningfully increased compared to those with other nanocomposite fillers [16]. The palm oil and methylester oils were more compatible with SBR due to the characteristic polarity of the oils. Also, this result indicates that the fillers with the hybrid oils resulted in stronger crosslinking with the SBR during vulcanization by enhancing the dispersion of fillers in the elastomer matrix. This explains the improved mechanical properties of the SBR nanocomposites shown in Figure 3. 

The physical properties of the SBR composites were examined, along with composites based on other embedded oils. Figure 3a shows the modulus and tensile strength of the SBR composites with different integrated oils. The modulus of the composites with OTG/M showed an increase of as much as 3% and 4% relative to that of OTG and OTG/D composites, respectively. This performance enhancement was due to the strong interfacial bonding and improved dispersion of the fillers within the elastomer matrix by the methylester with small molecular weight. Also, it was found that the equilibrium density results of the investigated vulcanization were promising and, consequently resulted in products that confirmed the other tested properties in Table 2.

Furthermore, the elongation at the break of the SBR with OTG/M (490%) shows it had enhanced properties compared to the SBR with OTG (460%), as shown in Figure 3b. The increased reinforcement can be attributed to enhanced interfacial adhesion between the fillers and elastomer, and restriction of the motion of the elastomer segmental chains by the improved dispersion of the fillers within the elastomer by the OTG/M. Figure 3c also shows a comparison of the toughness of the SBR composites compared to those of other oil composites. The toughness (area under the stress–strain curve) of the SBR/OTG and the SBR/OTG/M nanocomposite were 6.71 and 10.01 MJ/m^3^, respectively; the SBR/OTG/M nanocomposite exhibited a toughening effect as high as 49%. The tensile strength, elongation and toughness of the SBR composite at 3 phr OTG/M loading was much higher than those of other composites. This indicates that an applied mechanical load may transfer to fillers through interfacial interactions and uniform dispersion [17]. 

In Figure 3d, the fatigue properties of the SBR composites were measured after 3000, 6000, 10,000 cycles. Fatigue crack growth (*d*c/*d*n; cm/cycle) was calculated with the following equation: [18,19,20,21,22,23]

*d*c/*d*n = A∙G^α^ (4)

where c is the crack length, n is the cycle numbers, and G is the tear energy. This equation means that fatigue crack growth is proportional to the tear energy, and Equation (4) indicates that when tearing energy is decreased, fatigue life is increased. The SBR/OTG/M sample exhibited a remarkable reduction in crack length, even after 10,000 cycles, and the fatigue crack growth of SBR/OTG/M (3.51) was decreased by over 53% compared to those of the SBR with OTG (5.37) in Table 3. Furthermore, the fatigue life of the OTG/M-embedded SBR was longer than those of the OTG composites, as shown in Table 3. The growth in the fatigue cracks of composites using OTG/M decreased because the fillers were uniformly dispersed in the SBR, due to the formation of a segregated network through the methylester with small molecular weight.

**Figure 4:**
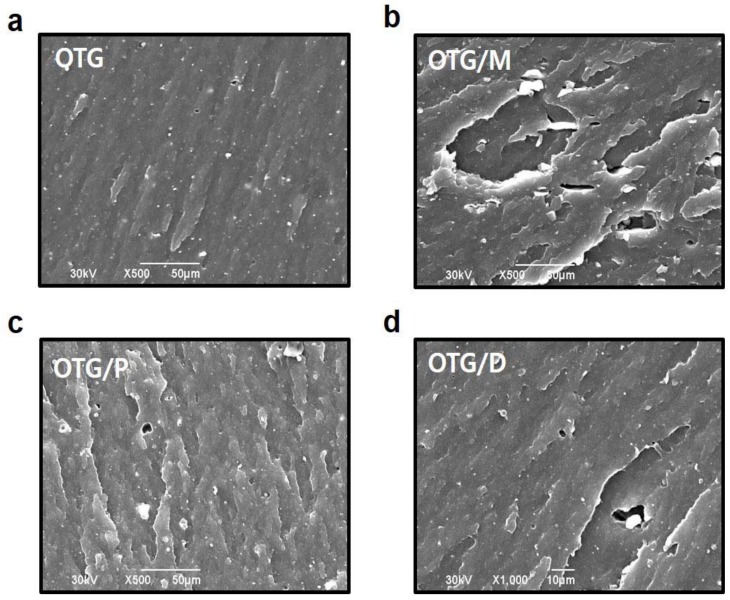
Scanning electron microscope (SEM) images of elastomer composites. (**a**) SEM images of the fracture surfaces of the elastomer composites with OTG. (**b**) SEM images of the fracture surfaces of the elastomer composites with OTG/M. (**c**) SEM images of the fracture surfaces of the elastomer composites with OTG/P. (**d**) SEM images of the fracture surfaces of the elastomer composites with OTG/D.

Scanning electron microscopy (SEM) provides direct evidence for evaluating the dispersion of fillers and phase morphology correlated with the mechanical properties of elastomer composites. The SEM images of the fracture elastomer composites are exhibited in Figure 4a–d. The SBR composites with OTG/M had a roughened fractured surface, indicating stronger interfacial adhesion between fillers and SBR matrix. Also, the fillers dispersed in the elastomer matrix are shown in the SEM images.

**Figure 5:**
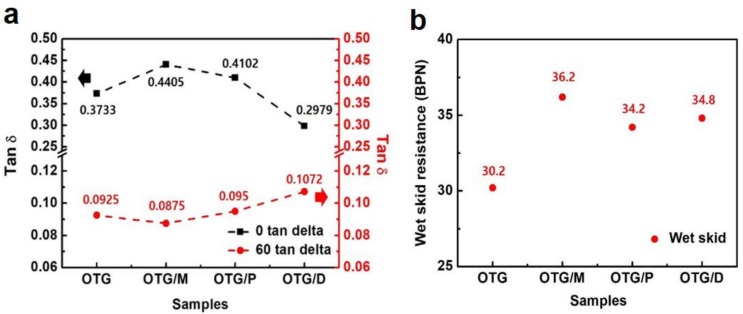
Dynamic properties of elastomer composites. (**a**) Tan δ of the elastomer composites with hybrid oils. (**b**) Wet skid resistance of the elastomer composites with hybrid oils.

Generally, dynamic mechanical properties are important because many engineering elastomers are subjected to dynamic loading. The dynamic mechanical properties (DMA) of elastomer composites, such as tan δ (tan δ is defined as the ratio of loss modulus to storage modulus) of the SBR composites with diverse oils versus temperature, respectively, are depicted in Figure 5a. The DMA test showed the resulting physical properties of the elastomer. This is often used to predict both rolling resistance and wet traction properties. With the addition of OTG/M, the tan δ of 0 °C was about 18% higher than that of SBR/OTG, as seen in Figure 5a, indicating pneumatic grip tire performance. Also, in Figure 5a, the 60 °C tan δ of the pneumatic tire with OTG/M was about 6% lower than that of the SBR with OTG. Generally, the lowering of tan δ at 60 °C correlates to an improved rolling resistance tire using OTG/M, which has great potential for improving wet performance. Also, the wet skid resistance result supports the DMA results of the elastomer composites in Figure 5b. The wet skid resistance of SBR/OTG/M exhibited a remarkable increase due to enhanced wet traction. Furthermore, these results are consistent with the mechanical properties of the composites in Figure 3.

**Figure 6:**
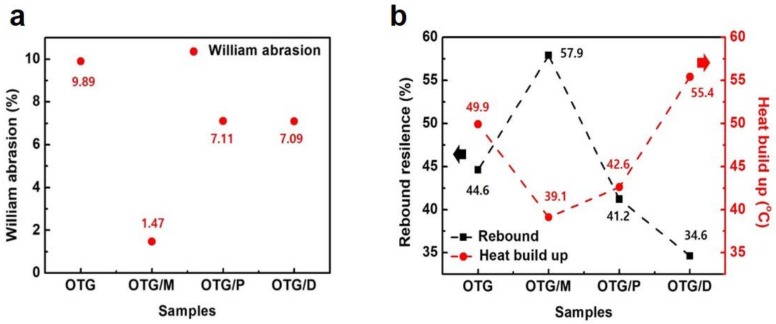
Abrasion, heat build-up properties of elastomer composites. (**a**) William abrasion of the elastomer nanocomposites with hybrid oils. (**b**) Rebound resilience and heat build-up of the elastomer composites with hybrid oils.

The abrasion properties of SBR composites with different oils are shown in Figure 6a. For the SBR composites with OTG/M, the William abrasion property was improved by enhancing the dispersion of fillers in the elastomer matrix. Furthermore, the abrasion of the composites using OTG/M was much lower than that of OTG, implying effective alleviation of heat build-up and the damping capability of the SBR systems. In Figure 6b, it was found that the SBR nanocomposites using OTG had the lowest heat build-up of 39.1 °C; the SBR/OTG/D nanocomposite with large molecules of dammar had a higher value of 55.4 °C, while the rebound properties of elastomer composites showed the opposite tendency. The heat build-up and rebound results also support the abrasion results of elastomer composites. 

The SBR/OTG/M composites homogeneously dispersed in the SBR matrix produced remarkable improvements in mechanical properties such as modulus, tensile strength, toughness and fatigue properties, even at low loadings, and these results are ascribed to the enhanced interfacial bonding and homogeneous dispersion between the elastomer matrix and fillers. Moreover, the incorporation of the OTG/M into the SBR matrix also significantly improved abrasion, heat build-up and rebound resilience properties due to the small molecular weight and similar polarity. Therefore, we can confirm that our approach provides a fascinating new potential for scalable and commercial polymer engineering.

## 4. Conclusions

In summary, we fabricated palm oil-based hybrid oils which were integrated into an elastomer matrix using a simple two-roll mixer mixing approach. Furthermore, we developed a novel approach of preparing elastomer composites with hybrid oils that produced improved mechanical and fatigue properties by improving interfacial interaction, using hybrid oils with small molecular weight and similar polarity. The results showed that the OTG/M were apparently more effective oils than OTG, OTG/P and OTG/D for producing elastomer composites with extraordinary synergetic effects at low loading levels. The newly developed hybrid oils and their utilization as a novel networked reinforcement may open up new opportunities for the preparation of elastomer composites with high performance.

## Figures and Tables

**Table 1:** Formulation of the elastomer composites.

Materials	OTG	OTG/M	OTG/P	OTG/D
Styrene butadiene rubber	100	100	100	100
Carbon black	50	50	50	50
Stearic acid	1	1	1	1
Palm oil	3	-	-	-
Palm oil and methylester	-	3	-	-
Palm oil and palm monoglyceride	-	-	3	-
Palm oil and dammar	-	-	-	3
Sulfur	1.75	1.75	1.75	1.75
ZnO	2	2	2	2
*N*-tert-butyl-2-benzothiazyl sulfonamide (TBBS)	1	1	1	1

**Table 2:** Swelling and crossing density characteristics of elastomer vulcanizates.

Materials	OTG	OTG/M	OTG/P	OTG/D
Equilibrium swelling (%)	151.55 ± 0.7	181.25 ± 0.55	172.29 ± 0.02	162.13 ± 0.09
Crosslinking density (mol/g·10^4^)	2.58 ± 0.6	3.19 ± 0.9	3.04 ± 0.7	2.68 ± 0.5

**Table 3:** Fatigue properties (fatigue crack growth and fatigue crack generation) of elastomer composites.

Materials	OTG	OTG/M	OTG/P	OTG/D
0	0	0	0	0
3000	2.45	1.11	2.51	2.31
6000	3.51	2.78	3.63	3.56
10,000	5.19	4.43	5.40	5.30
Generation	17,000	41,000	21,000	18,000
*d*c/*d*n	5.37	3.51	5.57	5.18
